# Platelet Microparticle Controversial Role in Cancer

**DOI:** 10.34172/apb.2021.005

**Published:** 2020-11-07

**Authors:** Mahnaz Nazari, Ehsan Javandoost, Mehdi Talebi, Aliakbar Movassaghpour, Masoud Soleimani

**Affiliations:** ^1^Hematology and Oncology Research Center, Tabriz University of Medical Sciences, Tabriz, Iran.; ^2^Student Research Committee, Tabriz University of Medical Sciences, Tabriz, Iran.; ^3^Department of Hematology, Faculty of Medical Sciences, Tarbiat Modares University, Tehran, Iran.; ^4^Department of Applied Cell Sciences, School of Advanced Medical Sciences, Tabriz University of Medical Sciences, Tabriz, Iran. Introduction

**Keywords:** PMP, Platelet, Microparticle, Cancer

## Abstract

Platelet-derived microparticles (PMPs) are a group of micrometer-scale extracellular vesicles released by platelets upon activation that are responsible for the majority of microvesicles found in plasma. PMPs’ physiological properties and functions have long been investigated by researchers. In this regard, a noticeable area of studies has been devoted to evaluating the potential roles and effects of PMPs on cancer progression. Clinical and experimental evidence conflictingly implicates supportive and suppressive functions for PMPs regarding cancer. Many of these functions could be deemed as a cornerstone for future considerations of PMPs usage in cancer targeted therapy. This review discusses what is currently known about PMPs and provides insights for new and possible research directions for further grasping the intricate interplay between PMPs and cancer.

## Introduction

Cancer is the first cause of death globally followed by cardiovascular diseases, imposing high costs on the health system. Following recent medical breakthroughs, the researchers’ focus has shifted toward tumorigenic mechanisms, cancer management, effective treatments and reducing treatment side effects. Improvement of the current treatment strategies requires a deeper understanding of the tumor microenvironment and its effective elements, which will ultimately lead to the use of combination therapies. Platelets as a blood component, are capable of playing a key role in tumorigenesis. In order to grow, tumors require a network of blood supply and the platelets floating within this network attach to the tumor cells, get activated, accumulate and might become part of the tumor microenvironment, potentially affecting parenchyma and tumor-dependent stroma.^1^ Increasing data are proving platelets as a key element bridging between thrombotic events and inflammatory pathways, leading to systemic inflammatory and immune processes.^2^ Not only platelets provide secreted, pleiotropic inflammatory mediators and factors orchestrating heterotypic interactions with endothelial cells, neutrophils, and monocytes, but they also produce microparticles.^3^ Microparticles (MPs) are a heterogeneous group of mainly spherical vesicles which contrary to exosomes, form through a process of membrane budding (exocytosis) and are basically present in all body fluids and maintained at a concentration of >10^6^/mL in blood under normal conditions, and reportedly increase during tissue hypoxia, oxidative stress, cell activation and a variety of diseases such as heparin-induced thrombocytopenia, thrombosis, idiopathic thrombocytopenic purpura, sickle cell disease, uremia, cancer, multiple sclerosis, rheumatoid arthritis, antiphospholipid syndrome and systemic lupus erythematosus.^4-6^ The first clues of a potential involvement of platelet MPs (PMPs) in cancer were provided when their high plasma levels were found in a variety of malignancies such as gastric and lung cancers, decreasing following therapy. Such findings are suggestive of a possible indicator of clinical prognosis.^7-9^ Initially, Chargaff and West^10^ identified PMPs as a precipitable factor in platelet-free plasma potentially capable of promoting thrombin generation. PMPs are currently known to comprise the majority of MP population in peripheral blood and account for over 70% of all extracellular vesicles.^11,12^

PMP characterization is generally based on electron and atomic microscopy, and analyzing protein markers, and single particle analyzers. Size distribution of PMPs varies in a wide range between 50 to 2000 nm, but is mainly within the 100-800 nm range.^13-15^

**Figure 1:**
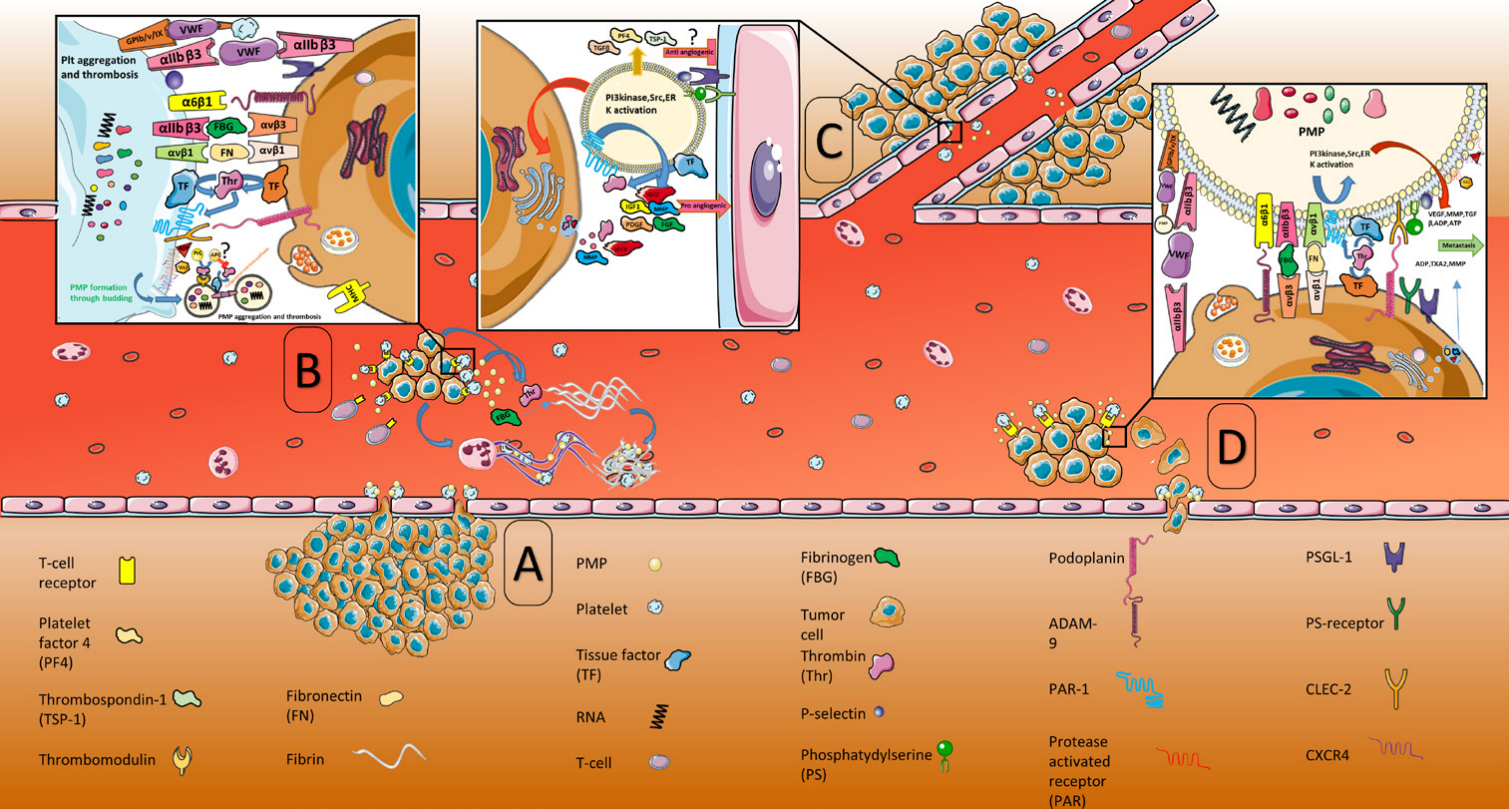
**PMPs in cancer progression.** **(A)** Endothelial dysfunction and poor coverage by pericytes lead to tumor cell intravasation. (**B)** Cancer cells promptly bind to platelets and activate them in bloodstream through adhesion molecules expressed on both cells. These interactions protect tumor cells against immune surveillance (platelet binding to tumor cells prevents tumor MHC molecule presentation to T-cells) and lead to the release of PMPs and activation of biomolecules participating in thrombosis, angiogenesis and metastasis. Tumor cell-PMP interactions increase the procoagulant activity of PMPs, providing a procoagulant membrane surface for thrombin activation and forming a prothrombinase complex which travels in the circulation, causing distant clot formation. PMPs can externalize tissue factor pathway inhibitor (TFPI) on their surface. The TF: FVIIa complex is regulated by TFPI, thrombomodulin, protein S or endothelial protein C receptor, conveying their ultimate participation in anticoagulant pathways. (**C)** Tumor cell-PMP interactions stimulate tumor cell expression of proangiogenic factors. The TF expression on PMPs initiates thrombin generation, subsequently boosting angiogenesis. PMPs may promote a switch to antiangiogenic state in endothelial cells. PMPs secrete PF4, TSP-1 and TGFβ which prompt a switch to anti-angiogenic state in endothelial cells. (**D**) Tumor cell-PMP interactions lead to metastasis. GPIIb/IIIa and TF play critical roles in metastases formation. GPIIb/IIIa supports stable adhesion of PMPs to tumor cells through binding to fibrinogen or vWF. GPIIb/IIIa (αIIbβ3) activation also initiates signaling which is responsible for the secretion of VEGF, ATP, ADP, TXA2, TGFβ and MMPs from PMPs. Presence of TF on the surface of PMPs provides a procoagulant membrane surface for thrombin generation, subsequently activating PAR-1 and signaling pathways mediated by PI3K, Src, and ERK, resulting in VEGF and MMPs secretion. PMPs also induce tumor cells to secrete ADP, TXA2 and MMPs. These mediators trigger tumor cell-induced platelet (and PMP) aggregation (TCIPA) which facilitates the microvascular arrest of tumor cells at distal sites during the metastasis process, tumor cell invasion, EMT and angiogenesis, all of which subsequently aid anchoring of metastatic tumor cells to distant sites, thereof assisting the establishment of new nodes. Moreover, ATP released from PMPs promotes tumor metastasis through relaxing endothelial barrier function upon binding the endothelial P2Y2 receptor and permitting extravasation of tumor cells. This figure was obtained using Servier Medical Art. (http://smart.servier.com/).

Means by which platelet microparticles get involved in tumorigenesis include shrouding tumor cells in circulation, allowing immune invasion, inducing a pro-coagulant state, aiding metastatic dissemination through establishing niches for the anchorage of circulating tumor cells, as well as anti-inflammatory, anticoagulant, antiangiogenic and apoptosis-inducing mechanisms (Figure 1). However, PMPs’ mechanisms of action after contacting the tumor cells is still a matter of debate.^16^ The present review will discuss how the PMPs influence tumorigenesis and their potential supportive and suppressive function in cancer progression.

## PMP

### PMP formation, structure and components

PMP formation through cell membrane budding is tightly linked to surface exposure of phosphatidylserine on platelets. Cell membrane phospholipids are asymmetrically arranged under physiological conditions; sphingomyelin (SM) and phosphatidylcholine (PC) are present in the outer layer while phosphatidylethanolamine (PE) and phosphatidylserine (PS) lie in the inner layer. Membrane asymmetry is controlled by the “flip-flop” mechanism which is regulated by phospholipid transporters including scramblase, flippase and floppase.^17^ flippase, directed toward the cytosol and ATP-dependent transporters; (ii) floppases, directed toward the extracellular environment and ATP-dependent transporters; and (iii) scramblases, bidirectional and ATP-independent transporters. Scramblases allow for a random distribution of lipids between the membrane bilayers. The flippases are very selective for PS, and their action is responsible for maintaining this phospholipid mainly sequestered at the inner leaflet of the cell membrane.^18,19^ Platelet activation by agonists such as collagen, ADP, thrombin, and Ca^2+^ ionophore, activates resting platelets and increases intracellular calcium, which in turn, inactivates flippase and induces floppase and scramblase activation, resulting in immediate exposure of negatively charged phospholipid PS.^17,20^ The energy required for this translocation is supplied from the ATP provided by Ca^2+^-dependent proteolysis degradation.^21^ As intracellular calcium flush occurs, cell blebbing can happen through cysteine protease and μ calpain, which in turn leads to disaggregation of cytoskeleton constituents, as well as α-actinin and talin. In platelets though, μ calpain inhibition is reported to prevent PMP shedding and its activation to be mediated by elevated cyclic AMP levels. As a result of activation of μ calpain and subsequently protein kinase A, cytoskeleton proteolytic degradation is triggered, which results in membrane blebbing and PMP release.^19^ Transmembrane protein 16F) TMEM16F) has been suggested to be necessary for phospholipid scrambling and PMP release.^22^ Some studies have been identified that there are some other specific molecular events to explain the connection between the increase of intracellular calcium after platelet activation and PS externalization. It has been suggested that PS externalization is another proposed cause of influx in the calcium channels, leading to calcium stores depletion, also known as store-operated calcium entry (SOCE), shown to be regulated by actin cytoskeleton. Through rearranging actin molecules of the cytoskeleton, GTPase Rho A acts in SOCE regulation and the subsequent PS exposure.^19,23^

While membrane scrambling and PS exposure are generally considered to be essential for PMP release, a considerable share of PMPs do not expose surface PS,However, we still cannot comprehend the intricacies of shedding of non-PS-exposing PMPs, implicating further research to grasp involved cellular mechanisms.^12,19^ Other processes which have also been implicated in PMP formation are proteasome function, protein tyrosine dephosphorylation, and calmodulin activation, the influence of which is not completely distinct.^12,24,25^ Detailed studies on PMPs have revealed noticeable complexity and heterogeneity of surface markers, content and size distribution.^25,26^ They are also different from megakaryocyte derived MPs based on surface markers. Table 1 presents the differences between platelet and megakaryocyte derived MPs. Platelet Microparticles are affected by the stimulus of their generation and their structural heterogeneity tightly depends on the mechanism resulting in their generation.^15,27^ Based on structural variations, PMPs can be categorized into three groups of single-layered PMPs, multi-vesicular PMPs, and organelle-containing PMPs. For instance, PMPs formed as a result of platelet stimulation by thrombin are comparatively smaller in size and contain cytoplasm, cellular components and organelles such as a mitochondrion, alpha- and glycogen granules.^15^

**Table 1:** Diversity of MPs markers based on cell of origin

**MP Source**	**Specific Markers**	**References**
Megakaryocyte	GPVI	^23,25,34^
	αIIbβ3 (CD41), CD42b	
	Filamin A	
Activated platelet	P-selectin(CD62P)	^23,25,35^
	LAMP-1	
	CD31, CD42b,CD36,CD61, αIIbβ3 (CD41)	
	PS+/-	
Apoptotic platelet	CD31, CD42b	^19,23,35^
	Histones	
	Fragmented DNA	
	High levels of phosphatidylserine	

Abbreviations: MP: Microparticle; GP: Glycoprotein; CD: Cluster of Differentiation; LAMP-1: Lysosomal-associated membrane protein 1; PS: Phosphatidylserine.

The density of PMPs largely depends on the quantity and quality of the glycoproteins, which mostly consist of membrane transporters and adhesion receptors. PMPs carry over forty different glycoproteins including IIb/IIIa, Ib/IX, P-selectin and gp53, as well as receptors for a number of coagulation factors. A variety of molecules are found in PMPs including coagulation, transcription and growth factors, enzymes, adhesion molecules, cytokines, chemokines, complement proteins, bioactive lipids, lipid mediators factors, apoptosis regulators and miRNAs.^28,29^ A comprehensive summary of PMP’s content and surface markers and their functions is outlined in Table 2. Many bioactive substances are released during platelet activation, which are typically stored in their α and dense granules. The fact that PMPs have a higher content of PS and P-selectin compared to their cell of origin, suggests either the existence of a dynamic process for content selection or that the PMPs arise from particular regions of platelet membrane rich in such factors, while the findings regarding flow-induced protrusions also promote the hypothesis that the budding might occur in specific regions.^30,31^ A proportion of PMPs may also transfer their mitochondria, while there are studies speculating the role of PMPs as a source of circulating nucleic acids. Size seems to be another factor influencing PMPs constitution, as size distribution and content have been revealed to be correlated with one another.^32,33^

**Table 2:** PMP’s content and surface markers and their functions

**PMP surface marker and content**	**Function**	**References**
CD42b (GPIb)	Adhesion to vWF	^36-38^
	Neutrophil activation	
CD62P (P-Selectin)	Binding to PSGL-1	^33,34,38-40^
CD42a (GPIbIX)	Adhesion	^41,42^
CD61 (GPIIIa)	Adhesion	^43-45^
	Aggregation	
CD41/61 (GPIIb/IIIa, αIIbβ3)	Adhesion	^46-48^
	Aggregation	
	Tumor cells metastasis	
	Binding to fibrinogen	
Lysosomal-associated membrane protein-3 (LAMP3, CD63, gp53)	Adhesion	^49^
	Inflammation	
Receptors for coagulation factors	Binding to FVa and FVIIIa	^33^
Anionic phospholipids	Passive procoagulant activity	^50^
MMPs	Degrading ECM	^11^
CXCR4	Inflammatory response	^47,51^
Cytokine receptors: TNFR-I, TNFR-II	TNFα-induced CD40L expression	^40,52^
TF	Activating coagulation	^50^
PAR-1 (protease-activated receptor)	Procoagulant activity	^47,53^
MHC1 (probable)	Presenting antigens to T cells	^54,55^
CD40L (CD154)	Activating B Cells	^33,56^
C-type lectin-like receptor (CLEC-2)	Probably binding to Podoplanin (PDPN)	^57^
LPC (probable)	Platelet activation, spread, aggregation and migration	^58^
	Vascular inflammation	
Complement activator (gC1qR , IgG)	Complement activation	^59^
	Inflammation	
Complement regulators (C1-INH, CD55, CD59)	Regulating complement system	^59^
Enzymes (cyclooxygenase-1,12-lipoxygenase, caspases 3 and 9, Heparanase, NO synthase)	Pro/anti-inflammatory response	^25,33,60,61^
	Apoptosis	
	Tumorgenesis	
Growth factors (VEGF, PDGF, TGFβ, bFGF, IGF1)	Angiogenesis	^33,62^
	Metastasis	
Cytokines (IL1b, IL-6, IL-8)	Inflammation	^33,62-64^
	Angiogenesis	
	Megakaryopoiesis	
Chemokines (CCL5, CCL23, CXCL7, CXCL4)	Monocytic arrest on ECs	^33,49,62,65^
	Negative angiogenesis factor	
	Suppressing neutrophil apoptosis	
S1P, AA, Thromboxane A2	AA delivery to cells	^65,66^
Transcription factors	Regulating inflammation and immunity	^33,67^
MicroRNAs	RNA transfer to target cells	^30,68,69^
Mitochondria	Producing inflammatory mediators	^25^
	Inducing leukocyte activation	
Thrombospondin	Antiangiogenesis factor	^70^
Platelet-activating factor	Activating neutrophils and macrophages	^71^

Abbreviations: PMP, Platelet microparticle; vWF, von Willebrand factor; PSGL-1, P-selectin glycoprotein ligand-1; ECM, Extracellular matrix; CXCR4, C-X-C chemokine receptor type 4; TNFR, Tumor necrosis factor receptor; MHC, major histocompatibility complex; FasL, Fas ligand; VEGF, Vascular endothelial growth factor; PDGF, Platelet derived growth factor; TGFβ, Transforming growth factor beta; bFGF, basic fibroblast growth factor; IGF1, Insulin-like growth factor 1; IL, interleukin; EC, Endothelial cell; AA, Arachidonic acid; S1P, Sphingosine-1-phosphate; MMPs, Metalloproteinases; TF, Tissue factor; LPC, Lysophosphatidylcholine.

### Platelet activation mechanisms

Factors triggering PMP formation in circulation include platelets apoptosis, platelets exposure to complement component C5b9, physiological or pathological elements resulting in cell activation (thrombin, collagen, ADP, Ca^2+^ ionophore) or infectious agents (e.g. bacterial lipopolysaccharide or influenza virus H1N1), shear stress, blood processing and storage (PMP counts double over 5 days of storage in apheresis concentrates) and mediators released by tumor cells.^25,72^ Dual stimulation with thrombin and collagen or a single agonist mixed with shear stress has been reported to cause maximal PMP production. For instance, the requirement of von Willebrand factor (vWF) for shear-generated PMP is supported by the evidence that antibodies blocking the vWF receptor (CD42b) inhibit PMP production.^73-76^

## PMPs function

### PMP and intercellular communication

Due to their potency as intercellular communication mediators, PMPs have recently been considered of particular interest by researchers. PMPs interact with many cells such as neutrophils, monocytes, endothelial and tumor cells to induce phenotypic changes or new functions in these cells by delivering a variety of factors including bioactive proteins, lipids, enzymes, surface receptors, growth factors, transcription factors and miRNAs and are even capable of transferring infectious agents such as HIV and prion. PMPs transfer CXCR4 receptor to cells lacking it and make them vulnerable to X4-HIV. Plasma and platelets account for the main source of cell-associated prion proteins in human blood. Studies alike also report this protein to be released by apheresis-obtained platelets.^1,30,77^ Although several poorly understood modes of interactions have been reported, three modes have been hypothesized through which the PMPs interact with the other cells. One is signaling proteins and bioactive lipids present on PMP surface stimulating the receptors on the target cells.^78^ Another hypothesis is the fusion of PMPs with the target cells to transfer the membrane integral proteins, while the third suggestion is PMP internalization and unpacking for the delivery of miRNAs and cytosolic enzymes.^16^ Membrane fusion between PMPs and cells leads to PMP content deposition in the recipient cell’s cytosol. This fusion process seems to be dependent on PS. However, PMPs find another way to interact with the target cell.^79^ Gas6 is a secretory protein which binds to the membrane PS and then functions as a ligand for tyrosine receptor kinases Axl, Tyro3, and MERtk.^80^ A recent study demonstrated that extracellular vesicles (including platelet microvesicle or PMP) are sorted into the endosomal pathway, moving quickly through the cytoplasm and then stalling at the endoplasmic reticulum, before eventually fusing with lysosomes for degradation inside the target cell.^81^ However, further studies are still required to clearly perceive the regulation of PMPs uptake. Mechanisms concerned in PMP adhesion as well as its internalization within tumor cells are yet to be elucidated, though are hypothesized to involve interactions with various receptors like GP1b, p-selectin and PS receptors on the surface of tumor cell, alongside other interactions such as phagocytosis or fusion.^82,83^

### PMP, inflammation and metastasis

Several mechanisms have been known for activated platelets to signal their target cells involved in inflammatory interactions, some of which occur through secreting mediators which might involve PMPs.^84^ PMPs are also reportedly increased in several other disease states with a recognized inflammatory component involved.^85-87^ Particular effects of PMP molecular transfer might be dependent on the type of target cells as well as the underlying inflammatory disease and certain patient factors.^88^ They might also exert anti-inflammatory effects, the mechanisms of which remain to be known. An overview of PMP involvement in inflammation is provided in Table 3.

**Table 3:** PMP involvement in inflammation

**PMP’s inflammatory mediator**	**Target cell**	**Outcome of interaction**	**References**
RANTES (CCL5)	Monocytes Activated endothelial cells	Vascular wall infiltration	^16,96^
		Stimulating chemotactic motility	
		Inducing monocytic arrest on endothelial cells	
AA	Monocyte – Endothelial cells	Activation of PKC	^97^
		Increased adherence between monocytes and endothelial cells	
		Increased chemotaxis of U-937 promonocytic cell line	
CD41 and CD62P	Endothelial cells	Endothelial cell activation	^11^
PS	Macrophages Dendritic cells	Neutralizing dendritic cells and macrophages phagocytic activity	^98^
Phosphatidylserine, GPIIb/IIIa, P-selectin	Monocytes	Activating monocytes	^99^
		Protumorigenic effect	
		Upregulating phagocyte markers expression	
PPARγ/RXR complex	THP-1 monocytic cell line	Aggregation of THP-1 cells	^100^
		Producing TF+ monocytic MVs	
		Modifying gene expression	
miR 126-3p	Macrophages	Inducing phagocytic phenotype	^101^
		Downregulation of cytokine/chemokine secretion	
		Induced monocyte differentiation to M2 macrophage	
PS?	Macrophages	Reduced release of TNF-ɑ and IL-10	^98^
		Immediately induced release of TGF-β from macrophage	
AA	U-937 (promonocytic cell line)	Increased Mac-1 and ITGAL (integrin subunit alpha L) expression	^66,102,103^
		Increased chemotaxis	
AA	Endothelial cells Monocytes	Expression of thromboxane A2 and COX-2 in endothelial cells	^66,102^
		Facilitating platelet aggregation	
Facilitating monocyte-EC interaction
Mitochondria	Leukocytes	Hydrolysis of mitochondrial membrane by sPLA2-IIA producing inflammatory mediators which promote leukocyte activation	^32,84^
CD154 (CD40 L)	B cells	Switch of antigen-specific IgG secretion	^56^
CD154 (CD40 L)	Monocytes	Increased inflammatory signals (IL1β, TNFα, MCP1)	^49,104,105^
		Stimulating monocyte-derived dendritic cells maturation	
miR-183	NK cells	Knockdown of NK activation adapter DAP12	^91,106,107^
		Suppressing NK cell inflammatory response to tumor	
TGFβ1	CD4+ T Cells	Increased TGFβ1 production	^108^
		Increased differentiation of CD4+ naive Tcells to FOXP3+ regulatory Tcells	
PF4(CXCL4)	CD4+ T Cells	Anti-inflammatory effect through reducing IFNγ, IL6, TNFα expression	^109^
PF4(CXCL4)	Treg cells	Treg stability in an inflammatory environment	^110^
		CXCR3-mediated signaling in activated T cells	
		Negative regulator of TH17 differentiation	
P-selectin	Treg cells	Treg stability in an inflammatory environment	^110^
		PMP adhesion to Tregs through PSGL-1	
		Prevention of peripheral blood–derived Tregs differentiation into IL-17– and IFN-γ producing cells	
P-Selectin	Neutrophils	Triggering neutrophil activation, aggregation and phagocytosis	^111^
		Inducing adhesion to the endothelium	
GPIbα	Neutrophils (β2 integrin Mac 1 (CD11b/CD18)	Neutrophil activation	^49,111^
GPIIb/IIIa receptors	Neutrophils	Transferring GPIIb/IIIA to neutrophils participating in NFkB activation of neutrophils	^112^
sPLA2-IIA and 12-lipoxygenase	Neutrophils	Promoting PMP internalization	^113,114^
		Enhancing inflammation	
β defensin 1	Neutrophils	NETosis (neutrophil extracellular traps) Cancer associated thrombosis	^49,115^
PMP-miRNAs	Neutrophils	Not clear	^16^
PMPs miRNAs released from collagen-activated platelets	Leukocytes	Stimulating cytokine responses	^115,116^
		Regulating cytokines release	
12-lipoxygenase	Mast cells	Negative inflammatory regulator	^60^

Abbreviations: PMP, platelet microparticles; N/A ,Not applicable; MVs, microvesicles; AA, Arachidonic acid; COX-2, cyclooxygenase-2; EC, Endothelial cell; sPLA2-IIA, secretory Phospholipase A2 group IIA; IgG, Immunoglobulin G; TNFα, tumor necrosis factor alpha; MCP1, monocyte chemoattractant protein-1; NK cells, Natural killer cells; IFNγ, Interferon gamma; Treg cells, T regulatory cells; TH, T helper cells.

Platelets assist cancer progression in a number of levels, especially at the late stages of primary tumors and metastasis.^89,90^ Formation of platelet–tumor-PMP aggregates, might facilitate tumor cells microvascular arrest at distal sites during the metastasis process.^91^ Distant metastasis needs tumor cells to undergo the following: crossing the vessel wall, remaining in circulation, angiogenesis, and ultimately proliferation at a new metastasis site.^7,92^ The interactions between tumor cells and platelets which lead to metastasis depend on platelet capacity to bind to the injured vascular endothelium, its capability of paracrine regulation of tumor cell growth and proliferation, and its ability to protect neoplastic cells in circulation against immune cells, and PMPs are likely to contribute to metastasis in a similar fashion.^11^ Bakewell et al. suggested that integrin β3 (heterodimer of αVβ3 and αIIbβ5) plays a critical role in metastasis, while platelet receptor (GP IIb/IIIa) antagonist serves as a protective factor against bone and other organs metastases.^93^ PMPs induced by certain breast cancer cell lines have been shown to strongly potentiate invasion and migration of these cells, though how PMPs bind to these cells remains a mystery and contrary to previous assumption that integrin αIIbβ3 and P selectin are involved in the process, it is now demonstrated that neither are. Such findings, suggest the existence of a positive feedback mechanism, by which cancer cells magnify their aggressiveness through PMP release induction.^94^ Tissue factor (TF) has been well proven to function in tumor growth, angiogenesis and metastasis. Thereby, it is not a surprising finding that its presence on the surface of PMPs facilitates metastasis.^11,93^ The role of metalloproteinases has been proven in advancing tumor invasion and angiogenesis. PMPs not only secrete metalloproteinases but also induce prostate cancer cells to do so. Furthermore, the procoagulant PMP surface aids anchoring of metastatic tumor cells to distant sites, establishing new nodes.^90,95^ PMPs also increase proliferation of A549 human lung carcinoma cell line, leading to expression of abnormal cyclin D2 and formation of distal lung metastases in mice 7. The delivery of PMP-coated cells into mice increases distal metastasis to the bone marrow and lung, compared to the control group only treated with the murine lewis lung carcinoma cells.^7,93^ Moreover, ATP generated by tumor-associated platelets in the process of PMP formation in blood promotes tumor metastasis through relaxing endothelial barrier function.^91^

### PMP and thrombosis

Cancer-related venous thromboembolism (VTE) was firstly described in the mid-19th century. Since ‎then, the ever-growing risk of VTE has been the subject of intense research. VTE is described as the ‎formation of blood clots in deeper veins of arm, leg, or groin that travel in circulation or lodging in ‎the lungs (Pulmonary Embolism). This phenomenon occurs in 15 to 20% of cancerous patients.^117,118^ ‎An increased level of platelet-, monocyte-, and endothelial-derived MPs are correlated with ‎thrombotic events occurred in arterial and venous vessels^42,119^ In cancer patients suffering from ‎VTE, the increased procoagulant activity of MPs including PMP is already observed at baseline, implying that it might be considered as a prognostic marker for VTE.^117,118^ Sinauridze et al applied two *in vitro* models (i.e., spatial clot formation and thrombin generation assays) to investigate PMP membranes enrichment with CD62, PS, and factor X binding sites. They reported that PMPs show a 100-fold greater specific procoagulant activity compared to activated platelets.^31^ In another study, Zhao et al. showed that PMP plasma levels correlate with procoagulant activity of colon cancer and increase along with the advancement of cancer stage.^8^ Furthermore, PMPs might prove a source of “blood-borne” TF inherited from platelets.^119^ As TF plays an important role in thrombosis and is promoted by tumor cells, the TF-bearing PMPs are of significance in tumor cell-induced platelet aggregation (TCIPA).^63^ Campello et al showed that patients with unprovoked VTE and those with various cancers with or without VTE have remarkably higher PMP and TF-MP levels compared to the controls.^120^ Tesselar et al examined TF co-expression with CD61 through confocal immunofluorescence microscopy. They reported that these TF positive-PMPs may be formed by the fusion of PMPs and malignant epithelial cell-derived MPs.^121^ In line with the above study, Hron et al. observed a significantly higher TF positive-PMP level in advanced colorectal cancer patients than that of healthy individuals. This result can be explained by the considerable increase in TF positive-PMPs. They also speculated that colorectal cancer cells might transfer the TFs onto PMPs.^122^ Another result of this study was the considerably higher PS content on PMPs compared with leukocyte-derived MPs, which justifies the direct relationship between D-dimer levels and TF positive-PMPs.^122^ A meta-analysis covering four cohorts and two case-control studies reports that TF-bearing MPs (including PMPs) are associated with a higher risk of VTE in cancer patients, particularly in patients of pancreatic cancer.^23,118^ Toth et al observed that CD62-positive PMP levels are highly associated with the level of prothrombin. Moreover, using electron microscopy, they found that the number of PMPs adherent to vWF is 3.5 times higher in breast cancer patients compared to controls. PMPs are the most copious source of MPs and demonstrate an increased number of vWF-binding receptors including integrin αIIbβ3 or GPIb, which may have a possible role in thrombosis.^123^ PMPs bear a wide range of surface receptors, including integrin GPIbα-IX-V receptor complex, GPIIb/IIIa, CXCR4, and P-selectin. As a result, they provide a procoagulant membrane surface for thrombin activation and forming a prothrombinase complex that travels in the circulation. Hence, distant clots are formed that often exhibits a procoagulant effect outlasting the activated platelets that generated them75. Morel et al^124^ showed that anionic phospholipids on PMPs surface induce accumulation of procoagulant and protein C anticoagulant enzyme complexes. Here, depending on the cell of origin, PMPs are able to expose tissue factor pathway inhibitor (TFPI), thrombomodulin, endothelial protein C receptor or protein S and lead to their ultimate participation in anticoagulant pathways.They are capable of facilitating FVa inhibition by activated protein C (APC) while APC, dependent on protein S, can inhibit coagulation on MPs.^75,125^

Under particular conditions, anticoagulant properties of PMPs have been proven as beneficial for their potential role in the progress of the anticoagulant process in cancer. For example, in early sepsis, they can retain APC as an inhibitor of VIIIa and Va factors.^125,126^ Several studies have revealed that CD41-positive PMPs can promote the generation of small amounts of thrombin. Thus, an anticoagulant process along with the protein C system may be activated by Va and VIIIa inactivation.^5,21^ Knowing CD41-positive PMPs can prompt the generation of minute amounts of thrombin. However, it is disputable whether PMPs are a cause or a result of thrombosis 30. In this regard, no data is available about the PMP-associated anticoagulant effect on cancer cells. Furthermore, it is not clear whether platelet activation and thrombocytosis are ultimately the causative agents or the result of tumor progression.^127^ Overall, PMPs which were once explained as inert “cellular dust” are thereby no less than “thrombotic dynamite”, specifically in the state of malignancy, while they show anticoagulant properties as well.^91^

### PMP and angiogenesis

Growth, tumorigenesis and metastasis all depend on abnormal angiogenesis, which is characterized by the new blood vessels forming capillaries to sustain an adequate level of oxygen delivery.^128^ This procedure is dependent on extracellular matrix degradation, disruption of cell-cell contact and the proliferation, migration and capillary tube forming of endothelial cells. Imbalance between many proangiogenic (signaling pathways and growth factors) and antiangiogenic factors (endostatin, angiostatin, thrombospondin-1) regulates angiogenesis. Among notable proangiogenic factors are vascular endothelial growth factor (VEGF), platelet-derived growth factor (PDGF), basic fibroblast growth factor (bFGF), insulin-like growth factor 1 (IGF-1), epidermal growth factor (EGF), transforming growth factor beta 1 (TGF-β-1), regulated on activation normal T-cell expressed and secreted (RANTES), matrix metalloproteinases (MMP1, MMP2 and MMP9), angiopoietins (1, 2 and 4) and cytokines such as interleukin 6 (IL-6) and interleukin8 (IL-8).^62,70^ All these pro- and antiangiogenic factors are secreted by platelets, tumor cells and PMPs, and take part in various stages of angiogenesis, including migration, proliferation and adhesion of endothelial cells.^129,130^ PMPs stimulate formation of network capillary tubes and stimulate tumor cell expression of proangiogenic factors.^7,78,84,131^ They are loaded with proangiogenic factors (PDGF, FGF, VEGF) released from α granules of the platelets of origin. Interaction of PMPs with endothelial cells may prompt a switch to a proangiogenic state, a phenomenon which could be extended by PMPs’ capacity to induce expression of kinase-dependent protein (MAPK p42/44 andAKT) and matrix metalloproteinase type 1 (MT1-MMP), as well as MMP-9,2 mRNA, interleukin 8 and VEGF in tumor cells.^132^ CXCR4 transfer to early outgrowth cells by PMPs amplifies the proangiogenic properties such as extracellular matrix adhesion or enhanced migration, proliferation and tube formation.^133^ The TF on PMPs initiates thrombin generation, resulting in VEGF secretion and prompting angiogenesis.^134^ Kim et al firstly demonstrated that PMPs raise *in vitro* proliferation, chemotactic migration and formation of capillary-like tubes of human umbilical vein endothelial cells (HUVECs). The fundamental mechanisms are depended on the protein growth factors such as FGF-2 and VEGF, and lipid growth factors such as S1P, all of which were inhibited by PI3K and Gi protein inhibitors.^78,128,134^ Prokopi et al demonstrated that PMPs can influence the angiogenic function of endothelial progenitor cells and endothelial tube formation is prompted by endothelial progenitor cell culture conditioned medium. Such outcome was reduced by PMPs removal from the conditioned medium by filtration, ultracentrifugation or prohibition of the platelet GPIIb-IIIa integrin complex formation.^131^ Studies show that ADP-mediated platelet activation induces VEGF release (not endostatin), while thromboxane A2 stimulates endostatin release but not VEGF. Platelets’ releasate generated by ADP-mediated activation, has been also shown to promote migration and formation of EC tubules in angiogenesis *in vitro* models.^84^ The proangiogenic influence of platelets and PMPs raises the question of what their mechanism is in light of angiogenic inhibitors presence besides the activators in platelets α granules. A possible explanation was proposed by Italiano et al who revealed various localizations of angiogenic cytokines among different granules.^135^ This hypothesis was further noted by another group who reported that the α granules are morphologically heterogeneous by 3D analysis and electron tomography.^136^

### PMP and apoptosis

Human platelets retain considerable quantities of FasL in their α-granules, which is either released into the medium or expressed on the surface once the platelet is activated.^137^ CD95 (Fas) expression is increased in cancer cells treated with platelets or its derivatives. This phenomenon could induce apoptosis in cancer cells through platelets interaction, which is in line with Bykovskaya and Yaftian et al findings.^138,139^ Although the presence of FasL on PMP surface has not been evidenced, the transmission of this receptor from platelets to PMPs is not far-fetched.^140,141^ sphingosine-1-phosphate which is a lipid component of PMP seems to mediate the anti-apoptotic effects of PMP on ECs.^84^ Human platelets also bear considerable amounts of CD40L in their alpha granules, which they either release to the medium or express on their surface once they are activated.^142^ CD40L expression on PMPs’ surface has been evidenced. The interaction between CD40L on platelets and PMPs’ surface with CD40 on pre-B ALL cells has also resulted in increased Fas expression in tumor cells which in turn induces apoptosis.^143,144^ Yet another study demonstrated that CD95L and CD95 possess several cancer related tumor-promoting and non-apoptotic functions, protecting and promoting cancer stem cells.^145^

### PMP and miRNAs

miRNAs are 22-nucleotide-long regulatory RNAs expressed in multicellular organisms. MiRNAs control most (>60%) of mammalian protein-coding genes.^146^ While some miRNAs are universally expressed, many are specific to tissue or developmental stage.^147^ The RNA-induced silencing complex (RISC) directed by the miRNA sequence leads to translational inhibition and mRNA degradation by Argonaute nucleases. miRNA role in gene expression is mostly fine-tuning and lowering noise in protein expression.^148^ Platelets are rich in pre-miRNAs as well as mature miRNAs. Platelet-derived miRNAs are packed into PMPs and account for a major share of platelet content released in PMPs.^16,28^ miRNA content of PMPs seems to form a subgroup of platelet miRNAs, suggesting active selection and incorporation of miRNAs into PMPs rather than simply random integration 68. Purified PMPs can regulate gene expression and transfer some miRNA content to cells such as leukocytes and endothelium following co-culture *in vitro.*^149-152^ New potentials of PMPs have recently begun to emerge, mainly presenting their capability to transfer miRNA content and regulate gene expression in target cells, which allows them to impact cancer development at different stages.^16^ Studies have proved that the content of circulating PMP miRNA is modified in different pathologies suggesting their potential as biomarkers for the disease along with platelet activation.^68,153^ Many miRNAs abundant in PMPs target both oncogenes and tumor suppressor genes in different cancers, and have been considered as prognostic markers for malignancies and implicated in therapy resistance. Similar to platelets, PMPs may be rich in variant isoforms of miRNA (isomiRs) with base-shifted seed sites.^82^ Next-generation sequencing of RNA expression as well as expanded mapping for miRNA targets are required to clarify the full extent of platelet miRNA impact.^16^ While PMPs have formerly been considered as cancer-promoting agents, their potential in transmission of miRNA and gene expression downregulation in different cell types implies the possible tumor-suppressive and apoptosis-inductive properties of PMPs.^16,82^ PMPs interaction with tumor cells in solid tumors via direct transfer of platelet-derived miRNAs also modulates tumor cell gene expression, resulting in tumor cell apoptosis, and inhibits growth of colon and lung carcinoma ectopic tumors, whereas miR-24 blockade in tumor cells accelerates tumor growth *in vivo.*^82^ In another study on the effect of PMPs on HUVECs, it was revealed that released PMPs after platelet thrombin-mediated activation are rich in miR-223. PMPs internalization by HUVECs and subsequent transmission of Argonaute 2-miR-223 complexes lead to downregulation of miR-223 targets inside the recipient endothelial cells, which might occasionally cause endothelial apoptosis.^82,83,150,154^ In presence of PMPs, anti-angiogenic modulators such as thrombospondin-1 (THBS-1) are substantially downregulated in HUVECs. Transfer of miRNA let-7a which targets THBS-1 in HUVECs, explains the neovascularization effect of PMP.^155,156^ Results of several studies emphasize the potential of PMP-mediated miRNA delivery to affect gene expression in target cells. Such findings provide unprecedented insight into mechanisms underlying horizontal RNA transfer and unveil several regulatory roles for PMP miRNAs in cancer progression. Platelet miRNAs transfer might also alter other sides of tumor biology including multi-drug resistance, known to be controlled by MPs.^157-160^ Thereby, platelets both positively and negatively affect cancer progression using different fashions and at several stages.^16,82,161^

Table 4 summarizes some studies on PMP miRNAs and their possible involvement in cancer fate.

**Table 4:** PMP’s miRNAs and their functions

**PMP miRNA(s)**	**Target cell**	**Physiological outcome**	**References**
miR-223	A549 human lung carcinoma cell line	Downregulation of EPB41L3	^152^
		Improvement of cell invasion	
miR-223	HUVEC	Downregulation of EFNA1 and FBXW7 RNA	^162^
miR-223	HUVEC	Downregulation of IGF-1R	^154^
		Apoptosis	
miRNA let-7a	HUVEC	Downregulation of thrombospondin-1 (THBS-1)	^155^
miR-22 miR-185 miR-320b miR-423-5p	HMEC-1 human microvascular endothelial cell line	Downregulation of ICAM-1	^151^
miR-126-3p	Macrophage	Downregulation of ATF3, ATP1B1, ATP9A and RAI14	^163^
		Downregulation of CCL4, CSF1, TNFα	
		Enhanced phagocytic capacity	
miR-24	Colon and lung carcinoma cells	Mitochondrial depolarization	^82^
		Increased caspase 3 activity	
		Induced apoptosis	
		Inhibited tumor growth	
miR-183	Natural killer cell	Suppressed cytolytic function of tumor-associated NK cell	^16^
miR-939	Epithelial ovarian cancer cell	Inducing epithelial to mesenchymal transition	^164^

Abbreviation: HUVEC, Human umbilical vein endothelial cell.

### PMP, tumorigenesis and clinical evidence 

Human cancer cells are able to promptly bind to platelets and activate them through α3-integrins on the cells or surface molecules such as glycoprotein IIb/IIIa on platelets, or via releasing mediators such as thromboxaneA2, ADP or tumor-associated proteinases.^121^ Such interactions result in increased expression of adhesion molecules, induced cytokine secretion, facilitated metastasis and angiogenesis, protection of tumor cells against immune surveillance, increased proliferation, migration and invasiveness of tumor cells, and activation of intracellular signaling pathways, wherein downstream signaling ultimately alters tumor cells reactivity with the endothelial cells.^129^ PMPs possess cytoplasmic proteins and chemokine receptors that strengthen tumor cell adhesion to endothelial cells, induce chemotaxis, upregulate matrix metalloproteinase production, and hence facilitate tumor cell invasiveness, which can also be prompted by the PS on the outer membrane of PMPs that coat tumor cells.^1,129^ Permeability of tumor neo-vasculature allows circulating PMPs immediate access to tumor cells, PMP infiltration, delivery of platelet-derived miRNAs to the tumor cells, growth suppression and gene regulatory effects, together expanding the reach and abilities of platelets and their microparticles to impact cancer progression beyond the intravascular area.^165^ Goldfinger et al noticed infiltration of PMPs in numerous solid tumor types and various tumor grades, though not in unaffected tissues. PMP exposure caused by vascular leak is hence likely limited to solid tumors distinguished from normal tissues.^82^ PMPs and platelets relationship with vascular leakage has recently been defined in ischemia and post-ischemic tissue repair, cardiovascular diseases, sepsis, diabetesand wound healing, indicating the impact of PMPs and miRNA transfer beyond the extent of solid tumor progression as a potential mediator of physiological responses to vascular leakage.^166-172^ Considering the critical role of platelets in cancer progression, PMPs could be involved in cancer cells proliferation, metastatic progression, inflammation, angiogenesis, apoptosis, immune evasion, extracellular matrix degradation, tumor growth and chemo-resistance.^173^ While an increased level of total PMPs in circulation has been observed in various types of malignancies such as gastric, ovarian, breast and lung cancer, high levels of PMPs have been correlated with tumor aggressiveness and poor clinical outcome.^172,173^ PMPs have been demonstrated to be capable of transferring CD41 to lung cancer cells, therefore triggering signaling molecules phosphorylation and promoting expression of MMPs and chemoinvasion. Moreover, PMPs which contain a certain epidermal growth factor receptor (EGFRvlll) typically expressed by gliomas cells, can transfer this oncogenic receptor to cancer cells lacking it, promoting their oncogenic activity.^174^ Nevertheless, Mege et al reported a decrease in PMP concentration in colorectal cancer patients compared to healthy individuals, which does not agree with Hron et al study^122^ showing higher TF positive-PMP levels in advanced colorectal cancer patients in comparison with healthy subjects.^174^

Independent of the tumor stage and under high shear stress conditions, platelet activation and aggregation as well as PMP formation are observed to increase. Although these increases vary by tumor type, they mostly appear to occur concurrently with cancer stage advancing and the highest levels are associated with advanced stages and distal metastasis of cancers.^175-179^

Table 5 outlines studies providing data on PMPs involvement in cancer.

**Table 5:** Clinical evidences of PMP involvement in tumorigenesis

**Cancer type**	**PMP level increase**	**PMP influence**	**References**
Myeloproliferative neoplasm	2 fold	Thromboinflammation	^180,181^
Colorectal cancer	Up to 4 fold	Lymph node metastasis	^122,176^
Breast cancer	3.5 fold	Improved cancer invasiveness	^7,11,123,182,183^
		Induction of angiogenesis and metastasis	
		Raised number of vWF-binding receptors	
		P-glycoprotein transfer to tumor cells	
		Induction of HER2	
Gastric cancer	Up to 35 fold in stage IV compared to stage I, II, III	Prediction of metastasis with sensitivity and specificity rates over 90%	^129,174,179^
		Plasma levels of PMPs higher in patients than healthy control	
Non-small cell Lung cancer	N/A	Induced expression of MMP9, MMP2 and angiogenic factors (VEGF, HGF, IL8)	^7,11,173^
		Activation of signaling molecules phosphorylation (MAPK p42/44 and AKY)	
		Induced chemoinvasion, adhesion to endothelium and fibrinogen, tumor progression, metastasis and angiogenesis	
Prostate cancer and HRPC	N/A	Increased adhesion of cancer cells to endothelium and ECM	^84,184,185^
		Assisted tumor invasion by increased metalloproteinases production and secretion	
		Increased cancer cells accumulation	
		Increased IL-8 secretion	
Neurogenerative disease	>N/A	Tumor development and metastasis	> ^186^
		Increased survival and proliferation of embryonic neural stem cells	
		Improved potential to differentiate to glia and neurons	

Abbreviations: PMP, Platelet microparticles; N/A, Not applicable; vWF, von Willebrand factor; HER2, Human epidermal growth factor receptor 2; MMP, Matrix metalloproteinase; VEGF, Vascular endothelial growth factor; HGF, hepatocyte growth factor; ECM, Extracellular matrix; HPRC, hormone-refractory prostate cancer

## Conclusion and Perspectives

In this review, we explain the activation, formation, component and structure of PMPs. Next, we describe their participation in cancer development. Despite the limited number of works on the role of PMPs in cancer, it is very important to characterize PMPs as a potential biomarker in cancer. The fluctuations of MPs during a cancer may suggest the significant role of microparticles as a cellular transporter, which plays a key role in cancer physiopathology. While PMPs involvement in metastasis and immune evasion of tumor cells are not fully understood, they have been demonstrated to mediate horizontal transfer of RNAs, leading to further ambiguities. Considering their ability to inhibit tumor growth, PMPs might counteract the platelets immediate impact on promoting cancer progression. However, findings are also suggestive of their indirect role in cancer promotion through certain platelet miRNAs’ transfer.^161^ Such contrasting data propose a possible dual-phase impact, whereby PMPs play anticancer roles in primary stages of tumor growth to encourage cancer progression mainly through miRNA-independent mechanisms.^16^ Further studies are required to comprehensively understand the interactions between PMPs and tumor cells influencing cancer progression. What appears to be undisputed thus far is that PMPs can serve as signaling molecules, passing on regulatory miRNAs to a variety of cells, yet tumor-specific miRNAs, as well as their target mRNAs, need to be determined alongside different phenotypic outcomes of mRNA silencing by tumor types. Inasmuch as circulating PMPs and their platelet-derived regulatory miRNAs are unequivocally involved throughout cancer progression, explicating concerned mechanisms will not only be of keen interest to researchers but will also represent a potential major breakthrough in cancer therapeutic targeting.

## Ethical Issues

Not applicable.

## Conflict of Interest

None.
